# Cardiac Involvement in Women With Pathogenic Dystrophin Gene Variants

**DOI:** 10.3389/fneur.2021.707838

**Published:** 2021-07-27

**Authors:** Tuva Å. Solheim, Freja Fornander, Anna A. Raja, Rasmus Møgelvang, Nanna S. Poulsen, Morten Dunø, Henning Bundgaard, John Vissing

**Affiliations:** ^1^Department of Neurology, Copenhagen Neuromuscular Center, Rigshospitalet, University of Copenhagen, Copenhagen, Denmark; ^2^Department of Cardiology, Rigshospitalet, University of Copenhagen, Copenhagen, Denmark; ^3^Department of Clinical Genetics, Rigshospitalet, University of Copenhagen, Copenhagen, Denmark

**Keywords:** dystrophinopathy, female carriers, cardiac MRI, echocardiography, cardiac, cardiac involvement

## Abstract

**Objective:** To determine the frequency and extent of cardiac involvement in female carriers of pathogenic variants in *DMD*, 53 women were examined through an observational, cross-sectional study.

**Methods:** Genetically verified female carriers of pathogenic *DMD* variants were examined by cardiac magnetic resonance imaging (CMR) with late gadolinium enhancement, echocardiography, 24-h Holter monitoring, ECG, and blood concentrations of skeletal and cardiac muscle biomarkers.

**Results:** Fifty-three female carriers of pathogenic *DMD* variants (mean age 49.6 years, 33 associated with DMD, and 20 with BMD) were included in the study. Sixty-two percent had cardiac dysfunction on echocardiography. On CMR, 49% had myocardial fibrosis, 35% had dilated left ventricles, and 10% had left ventricular hypertrophy. ECGs were abnormal in 72%, and abnormal Holter monitoring was found in 43%. Age did not correlate with myocardial fibrosis or cardiac dysfunction. Myocardial fibrosis was more frequent in carriers of pathogenic variants associated with DMD vs. BMD (61 vs. 28%, *p* = 0.02).

**Conclusion:** This study shows that cardiac involvement, affecting both structure and function of the heart, is found in over 2/3 of women with a pathogenic *DMD* variant. The study supports early cardiac screening, including ECG, Holter, and cardiac imaging, in this group of carriers, so that symptoms related to pathogenic variants in *DMD* can be recognized, and relevant treatment can be initiated. Longitudinal studies are needed to assess morbidity and mortality related to single, pathogenic *DMD* variants in women.

## Introduction

Duchenne (DMD) and Becker (BMD) muscular dystrophies are X-linked recessive disorders caused by pathogenic variants in the dystrophin gene (*DMD*). In affected males, the pathogenic variants cause progressive muscular wasting and weakness, respiratory failure, dilated cardiomyopathy, arrhythmias, and congestive heart failure (1, 2).

About 2/3 of affected males inherit the disease from their mother (3), 1/3 have *de novo* pathogenic variants. Female carriers may pass on the pathogenic variant to their daughters, resulting in a significant number of female carriers of pathogenic *DMD* variants. Female carriers were previously considered non-manifesting, but in the last decades, it has become evident that these women can be affected similar to, but milder, than affected males, and this includes cardiac involvement. The literature on cardiac involvement in female carriers is based on studies with one or two cardiac modalities, CMR studies in smaller patient populations, some retrospective studies (4–6), and some studies without genetic confirmation. Previous studies report a large span in the frequency of cardiomyopathy in female carriers, ranging from 3 to 84% (4, 7–13). This high variability may be related to the abovementioned limitations in the studies, but also to variable definitions of cardiomyopathy.

Early detection of cardiac involvement in dystrophinopathies is crucial for initiating treatment within the window of early interventions to potentially reduce cardiac symptoms and progression (14, 15). Therefore, knowing the extent of cardiac involvement in this population of carriers is paramount (16).

In this observational, cross-sectional study, female carriers of DMD/BMD were comprehensively examined to establish the frequency and extent of cardiac involvement.

## Materials and Methods

### Study Design

This cross-sectional study was conducted prospectively at the Departments of Neurology and Cardiology at the National Hospital, Rigshospitalet, in Copenhagen, Denmark, and was approved by the Danish Data Protection Agency and the National Committee of Health Research Ethics (H-16035677). Written informed consent was obtained for all participants, according to the Declaration of Helsinki. Participants were evaluated by medical history, transthoracic echocardiography, Holter monitoring (24 h), 12-lead ECG, and cardiac magnetic resonance imaging (CMR) with late gadolinium enhancement (LGE). Blood concentrations of creatine kinase (CK), creatine kinase MB (CK-MB), myoglobin, pro-brain natriuretic peptide (proBNP), and troponin T (TnT) were determined. A CK-MB/CK ratio was calculated for CK-MB values above the reference range and was considered suggestive of cardiac involvement, not skeletal muscle affection, when the CK-MB/CK ratio was ≥3%. Furthermore, manual testing of muscle strength was performed with the Medical Research Council scale for muscle strength (MRC). All participants also underwent lower extremity muscle MRI and strength investigations presented in the accompanying article in Frontiers in Neurology.

### Study Population

Subjects were recruited from our Neuromuscular Clinic (*n* = 3) or via the DMD/BMD carrier register (*n* = 50) at the Department of Clinical Genetics, Rigshospitalet. Inclusion criteria were as follows: confirmed pathogenic variant in the *DMD* gene, and a woman 18 years of age or older. Subjects were excluded if they had contraindications against CMR. One hundred and eight invitation letters were sent out, and in case of no response, they were followed up by a phone call. Fifty-three subjects who accepted the invitation fulfilled the inclusion and none of the exclusion criteria and were included in the study from December 2016 to April 2018. The remaining 58 invited subjects were either excluded on the basis of the exclusion criteria, did not wish to participate, or did not respond to neither letter nor phone call. Twenty of the 58 non-participating persons were interviewed by phone after the 53 included participants had been tested. This was done to uncover the presence of cardiac and skeletal muscle symptoms and their reasons not to participate, to assess potential bias in the selection of included subjects. The remaining 38 persons who did not participate could not be reached.

### Electrocardiography

A 12-lead ECG was performed using routine ECG recordings (GE healthcare MAC 3500, Freiburg, Germany). All ECGs were analyzed by TS and HB. An ECG was defined as abnormal in the presence of atrial flutter/fibrillation (AFL/AF), atrioventricular block (AVB) grades I–III, QRS-interval >120 ms, prolonged QTc >470, or incomplete right bundle branch block (IRBBB). Additionally, ECGs were assessed with special focus on ECG abnormalities known to be observed in DMD patients and carriers, which included increased R-wave in V1–V2 (>4 mm), increased R/S ratio in V1 or V2 in the absence of incomplete or complete right bundle branch block (RBBB), pathological Q-waves (>0.2 mV) in lateral (I, AVL, V6) or inferior leads (II, III, AVF), complete or incomplete left bundle branch block (LBBB/ILBBB) or complete RBBB (9).

### Holter Monitoring

A 24-h Holter was performed using a three-electrode PocketECG (Vicare, Warsawa, Polen). All holter monitor data were analyzed by TS and HB. Holter monitor was defined as abnormal in the presence of AVB grades I–III, AFL/AF, other supraventricular tachyarrhythmias (SVT) [>30 supraventricular premature contractions (SVPC) per hour or runs of ≥20 SVPC], frequent ventricular premature contractions (VPC) (≥30/h), non-sustained ventricular tachycardia (NSVT) (minimum of 3 beats at ≥100 bpm) and supraventricular or ventricular couplets and/or triplets (17).

### Transthoracic Echocardiography

Echocardiography was obtained with Philips EPIQ 7C (Philips Medical Systems, Best, the Netherlands) or Vivid E95 (GE Healthcare Vingmed Ultrasound AS, Horten, Norway) and analyzed by an experienced cardiologist using Philips IntelliSpace Cardiovascular version 1.2/QLAB version 10.5 or GE Healthcare EchoPac version 202, respectively, in accordance with current guidelines (18). Systolic dysfunction was defined as left ventricular ejection fraction (LVEF) < 54% and/or absolute global longitudinal strain (GLS) ≤ 20% (19). Diastolic function was classified according to current guidelines into normal or grades 1–3 (20). We defined diastolic dysfunction as grades 1–3 in persons <65 years and grades 2–3 in persons ≥65 years of age. Cardiac dysfunction by echocardiography was defined as the presence of systolic and/or diastolic dysfunction.

### Cardiovascular Magnetic Resonance

CMR was performed on a 3.0 Tesla MR scanner (MAGNETOM Verio Siemens, Erlangen, Germany) using a body matrix coil. All CMR images were analyzed by TS and AAR. Steady-state free precession end-tidal breath hold cine images with retrospective ECG gating were obtained in two-, three-, and four-chamber views, followed by a short-axis stack and a transversal stack, both covering the entire heart (8-mm slice thickness, no interpolated gap). T1 mapping was performed using the modified look-locker inversion recovery technique (MOLLI) on one mid-ventricular short-axis image. LGE was assessed by using T1-weighted gradient echo sequences in two-, three-, and four-chamber views and a short-axis stack contiguously covering the left ventricle. LGE and post contrast MOLLI images were obtained 10 min after administration of an intravenous bolus of gadolinium (0.15 mmol/kg body weight) (Gadovist, Bayer Schering, Germany). To exclude artifacts, cross-sectional views were acquired when LGE was present on standard views. Inversion time was 250–350 ms (manually adjusted to optimally null the myocardium).

CMR analysis was performed with CVI^42^ (Circle Cardiovascular Imaging, Inc., Calgary, Canada). Endocardial boarders of the left ventricle were traced on the short-axis cine images in the end-diastolic and end-systolic phase, respectively, and epicardial boarders were traced in the end-diastolic phase. The papillary muscles were incorporated as part of the cavity. End diastole and end systole were identified as the largest and smallest volume, respectively. Left ventricular (LV) mass was calculated by the software as the volume between the two borders multiplied by the myocardial density (1.05 g/cm^3^), excluding papillary muscles.

For the right ventricle, the endocardial borders were traced in the end-diastolic and end-systolic phase from the transversal stack; the papillary muscles were incorporated as part of the cavity. Endocardial borders of the left atrium were manually traced in ventricular end systole from the transversal stack. Left atrial appendage was included, but pulmonary veins were excluded from the atrial volume.

LGE images were first reviewed for visible LGE (areas with relatively increased signal intensity following administration of gadolinium contrast), and if positive, quantified. LGE was interpreted as present only if confirmed on both short-axis view and a matching location on long-axis or cross-sectional views. Quantification was performed as a signal intensity threshold of >6 SD above remote myocardium. The extent of dense replacement fibrosis was quantified as mass and as a percentage of total left ventricular mass. Manual correction was performed for obvious threshold errors.

MOLLI images where analyzed to construct T1 maps, recovery curves, and DICOM maps, both pre- and post-contrast. Extracellular volume (ECV) was calculated by the Equation (21):

$$\begin{array}{l} ECV=\\ (1-hematocrit)\;\frac{\frac{1}{myocardial\;post\;contrast\;T1}-\frac{1}{myocardial\;native\;T1}}{\frac{1}{blood\;post\;contrast\;T1}-\frac{1}{blood\;native\;T1}} \end{array}$$

An abnormal CMR was defined after current guidelines as LVEF <56%, LV end diastolic volume >143 ml/m^2^, LV mass indexed >74 g/m^2^, right ventricular EF <49%, right ventricular volume indexed >153 ml/m^2^ (22), left atrium max volume indexed >53 ml/m^2^ (23), the presence of late gadolinium enhancement, elevated ECV, or prolonged T1 native times above two standard deviations from normal values (ECV > 0.34, T1 native times >1,404 ms) (24).

### Statistical Analysis

Data were analyzed with Microsoft Excel and R Studio. Normally distributed values are presented as means ± standard deviation (SD) and data with skewed distribution as median (range). Categorical values are presented as frequency count (percentage), and differences among groups were analyzed using chi-square test and Fishers exact test. Comparison between categories was calculated with Mann–Whitney *U*-test. Correlation analyses were performed with Spearman correlation. The level of statistical significance was set to 0.05.

### Data Availability

Anonymized data will be shared with any qualified investigator on reasonable request.

## Results

### Study Population

The 53 women with pathogenic *DMD* variants originated from 45 families and were on average 49.6 years old (range: 26–81 years). Cardiac risk factors and comorbidities are listed in Table 1. About one-third of the participants complained about cardiac-related symptoms, mainly fatigue and/or dyspnea. Additional heart-related symptoms and cardiac medications are listed in Table 1.

**Table 1:** Baseline demographics for the 53 participating women.

Age (years), mean (SD)	49.6 (13.0)
BMI (kg/m^2^), mean (SD)	26.3 (5.27)
Heart rate (bpm), mean (SD)	71 (10.4)
Hypertension, *n* (%)	8 (15)
Diabetes mellitus, *n* (%)	1 (2)
Hypercholesterolemia, *n* (%)	7 (13)
Smokers, *n* (%)	20 (38)
Familial disposition to cardiac disease^*^, *n* (%)	23 (43)
**Cardiac medications**	
Beta-blocker, *n* (%)	2 (4)
ACE/Angiotensin II-inhibitior, *n* (%)	6 (11)
Diuretics, *n* (%)	7 (13)
Acetylsalicylic acid, *n* (%)	2 (4)
**Reported cardiac symptoms**	
Dyspnea, *n* (%)	8 (15)
Heart palpitations, *n* (%)	6 (11)
Fatigue, *n* (%)	15 (28)
Lower extremity edema, *n* (%)	4 (8)
Syncope or near-syncope, *n* (%)	3 (6)
NYHA II, *n* (%)	9 (16)
Chest pain, *n* (%)	0 (0)

* *Defined as first- or second-degree relatives with cardiac disease, including arrhythmias, congestive heart failure, acute myocardial infarction or valvular dysfunction*.

*BMI, body mass index; bmp, beats per minute; NYHA, New York Heart Association*.

### Interview With Non-participating Individuals to Assess Selection Bias

Of the 20 non-participants that were interviewed, 10 reported that they did not participate due to distance to the hospital and lack of time, six had too many hospital visits with their DMD sons or due to other diseases of their own that were unrelated to cardiac and neurologic disease. Three had not received the letter of invitation. The last wished to participate but had contraindications against MRI. None of the 20 individuals declined participation due to cardiac symptoms or muscle symptoms. There was no difference in symptoms reported by the non-participants compared with the participants: dyspnea (17 vs. 15%), fatigue (28 vs. 28%), palpitations (13 vs. 11%), lower extremity edema (8 vs. 6%), and syncope (6 vs. 6%).

### Circulating Biomarkers and Muscle Strength Testing

Over two-thirds of participants had elevated levels of skeletal muscle biomarkers of muscle damage (Table 2). Half had elevated levels of CK-MB, but none with a CK-MB/CK ratio ≥3%. Cardiac biomarkers (TnT and ProBNP) were elevated in <10%. The presence of cardiac fibrosis was associated with higher levels of myoglobin (*p* = 0.02), CK-MB (*p* = 0.02), and CK (*p* = 0.01), but not with higher levels of TnT (*p* = 0.35) or ProBNP (*p* = 0.23). We found a trend toward higher levels of CK and CK-MB in women with a pathogenic variant associated with DMD vs. BMD (CK *p* = 0.058, CK-MB *p* = 0.099).

**Table 2:** Skeletal and heart muscle biomarkers.

	**Median (range)**
Myoglobin μg/L (ref. 19–49 μg/L)	79 (34–873)
Creatine kinase (ref. 35–210 U/L)	246 (66–1,650)
Creatine kinase MB (ref. <4.0 μg/L)	3.8 (1.3–18.1)
CK-MB/CK ratio^*^ (ref. <3%)	1.68 (0.88–2.65)
Troponin T (ref. <14 ng/L)	(≤13–51)
ProBNP (ref. according to age^**^)	(≤5.9–73)
**Abnormal biomarker values**	**Number (%)**
Myoglobin>49 μg/L, *n* (%)	39 (74)
Creatine kinase>210, *n* (%)	30 (57)
Creatine kinase MB>4 μg/L	26 (49)
CK-MB/CK ratio≥3%	0 (0)
Troponin T ≥ 14 ng/L	5 (9)
ProBNP >ref. according to age^**^	3 (6)

* *CK MB/CK ratio was only calculated for CK-MB values ≥4.0 μg/L*.

** *18–44 år: <15.3 pmol/L, 45–54 år: <29.4 pmol/L, 55–64 år: <33.9 pmol/L, 65–74 år: <35.5 pmol/L, 75–125 år: <87.1 pmol/L*.

*CK, creatine kinase; ProBNP, pro-brain natriuretic peptide*.

The majority of participants (92%) had normal muscle strength when manual testing was performed. Four participants (8%) scored four or four minus on MRC in one or more of the tested muscle groups. Of these four, none had abnormal LVEF on echocardiography, and one had myocardial fibrosis on CMR. There was no association between cardiac affection and muscle weakness.

### Electrocardiography

Over half of the participants had at least one DMD-specific ECG change, as defined in the previous *Electrocardiography* section, largely due to high R waves in V1 and/or V2 (Table 3). Twenty-six percent had three or more DMD-specific changes. The most predominant non-DMD-specific abnormality was incomplete right bundle branch block, found in 15%. In total, 72% of the patients had one or more ECG abnormalities.

**Table 3:** ECG findings in 53 women with pathogenic *DMD* variants.

**General ECG measurements**	**Mean (SD)**
PR interval (ms)	146.7 (21.9)
QRS interval (ms)	90.8 (12.8)
QT (ms)	401.0 (28.7)
QTc interval (ms)	422.5 (21.2)
**DMD-specific abnormalities**	**Number (%)**
R >4 mm V1	12 (23)
R >4 mm V2	32 (60)
R/S >1 in V1^*^	1 (2)
R/S >1 in V2^*^	3 (6)
Q in lateral leads >0.2 mV	1 (2)
Q in inferior leads >0.2 mV	1 (2)
RBBB	1 (2)
LBBB	1 (2)
Any DMD-specific change	33 (62)
**Other measurements**	**Number (%)**
IRBBB	8 (15)
Left ventricular hypertrophy	1 (2)
Right ventricular strain	4 (8)
Any ECG change	38 (72)

* *In the absence of complete or incomplete right bundle branch block*.

*RBBB, right bundle branch block; LBBB, left bundle branch block; IRBBB, incomplete right bundle branch block; DMD, Duchenne muscular dystrophy*.

No significant association was found between the presence of DMD-specific changes on ECG and reduced LVEF, diastolic dysfunction, or LV dilatation on echocardiography, SVT, or frequent VPCs on Holter monitoring, IRBBB on ECG, or fibrosis on CMR.

**Figure 1:**
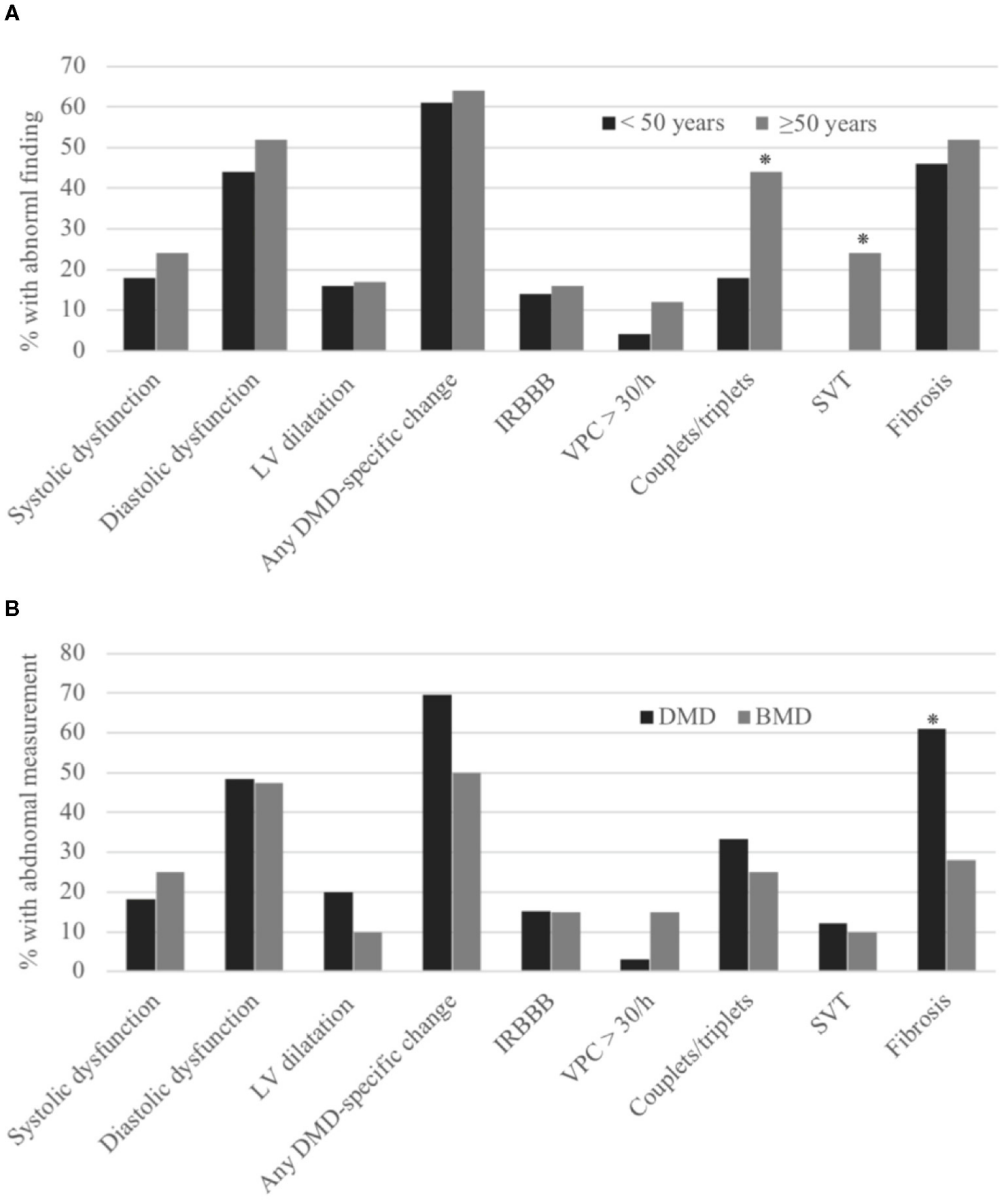
Cardiac abnormalities in women with pathogenic *DMD* variants. Comparing women younger or older than 50 years **(A)** and women with pathogenic *DMD* variants predicted to cause Duchenne muscular dystrophy (DMD) and Becker muscular dystrophy (BMD) **(B)**. LV, left ventricle; IRBBB, incomplete right bundle branch block, VPC, ventricular premature contractions; SVT, supraventricular tachyarrhythmias. *p < 0.05.

When comparing participants older or younger than 50 years, there was no significant difference in the incidence of IRBBB or DMD-specific changes (Figure 1A).

### Holter Monitoring

Four participants had frequent ventricular premature contractions. Six had supraventricular tachycardia, as defined in the previous *Holter monitoring* section. NSVT was found in two participants. Finally, supraventricular or ventricular couplets and/or triplets were seen in 17 participants. In total, 43% of the patients had abnormal findings on their 24-h Holter monitoring.

When comparing participants older or younger than 50 years, the incidence of couplets and/or triplets, and SVT was significantly higher in the participants older than 50 years. There was no significant difference in the incidence of frequent VPCs (Figure 1A). One participant had only 9.5 h of Holter monitoring due to a lack of compliance.

### Transthoracic Echocardiography

Systolic dysfunction was found in 33 (62%) participants, either as reduced longitudinal function in GLS and/or reduced LVEF. Twenty-seven participants (52%) had reduced diastolic function, of whom two (4%) showed a pattern consistent with restrictive filling (Grade 3). Structural changes in the left ventricles were found in <20%, predominantly as LV dilation (Table 4). Significant valvopathy was not found in any of the participants, although 21% had mild mitral insufficiency, 5% had mild tricuspid insufficiency, 5% had mild aortic insufficiency, and 4% had mild pulmonary insufficiency. Cardiac dysfunction on echocardiography, defined as the presence of systolic and/or diastolic dysfunction, was found in 33 (62%) of the participants.

**Table 4:** Echocardiography abnormalities in 53 women with *DMD* gene aberrations.

**Structural changes**	**Number (%)**
LVEDV indexed ≥62 ml /m^2^	8 (17)
LV mass >95 g/m^2^	1(2)
**Systolic function**	
EF (biplane) <54%	11 (21)
GLS ≤ 20%	29 (63)
**Diastolic function**	
LA volume indexed >34 ml/m^2^	9 (17)
Normal	21 (40)
Grade 1	25 (48)
Grade 2	0 (0)
Grade 3	2 (4)

*LVEDV, left ventricular end diastolic volume; LV, left ventricular; EF, ejection fraction; GLS, global longitudinal strain; LA, left atrium; indexed, divided by body surface area*.

No association was found between an abnormal echocardiography and the cardiac measurements compared in the *Electrocardiography* section. When comparing participants older and younger than 50 years, there was no significant difference in the occurrence of EF < 54%, diastolic dysfunction, or LV dilation (Figure 1A). Age correlated negatively with GLS (Spearman correlation *r* = −0.42, *p* = 0.003) but did not correlate with LVEF (*r* = −0.07, *p* = 0.6) or LV end diastolic volume (*r* = −0.2, *p* = 0.18).

### Cardiac Magnetic Resonance Imaging

Fifty-two patients completed CMR for volumetric measurements. One participant was not examined with CMR due to claustrophobia. Forty-nine patients had post-contrast imaging performed. The remaining three participants did not wish to be injected with the gadolinium contrast.

**Figure 2:**
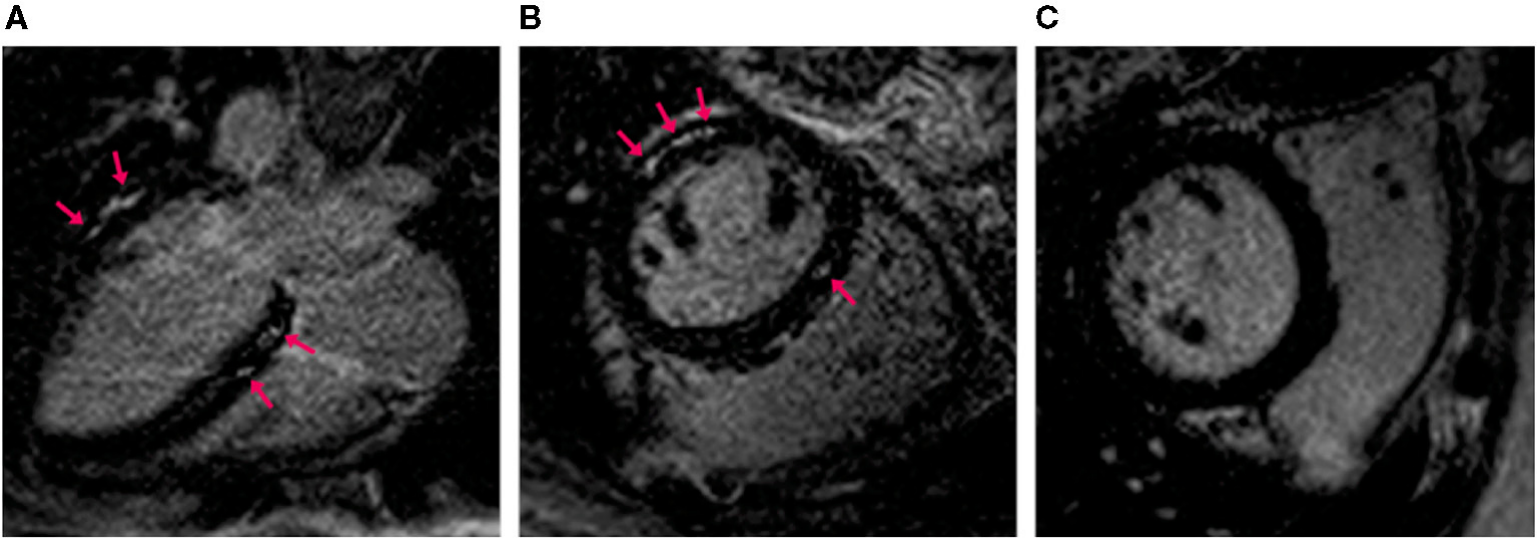
Magnetic resonance images of the heart with late gadolinium enhancement. Long axis **(A)** and short axis **(B,C)** images. **(A,B)** show myocardial fibrosis (arrows) in a 46-year-old woman. **(C)** shows the corresponding findings in a 42-year-old unaffected woman.

Myocardial fibrosis of the left ventricle was found in 49% of participants, with a median LGE quantity of 3.8% of total left ventricular mass (range 0.1–17.9%) (Figure 2). Eleven (24%) participants had elevated ECV on MOLLI images, and 55% of them also had myocardial fibrosis on LGE images. Only one (1.9%) had increased native T1 time.

Six participants (12%) had reduced LVEF (Table 5). Dilated left ventricles were found in 35%, and 10% had left ventricular hypertrophy. Three participants (6%) had dilated right ventricles and reduced right ventricular ejection fraction. Increased left atrial volume was found in 34%.

**Table 5:** Cardiac MRI abnormalities in 52 women with pathogenic *DMD* variants.

**Left ventricle**	**Number (%)**
LVEF decreased mildly (40**–**56%)	6 (12)
LVEDV increased mildly (143**–**179 ml)	16 (31)
LVEDV increased moderately (180**–**220 ml)	3 (6)
LVEDV indexed increased mildly (78**–**100 ml/m^2^)	18 (35)
LV mass indexed increased mildly (60**–**74 g/m^2^)	4 (8)
LV mass indexed increased moderately (75**–**90 g/m^2^)	1 (2)
**Right ventricle**	
RVEF decreased mildly (37**–**49%)	3 (6)
RV volume indexed increased mildly (104**–**153 ml/m^2^)	3 (6)
**Left atrium**	
LA max volume indexed increased (>53 ml/m^2^)	17 (34)
**Fibrosis**	
Any late gadolinium enhancement	24 (49)
ECV increased in MOLLI images (>34%)	11 (24)

*LVEF, left ventricular ejection fraction; LVEDV, left ventricular end diastolic volume; LV, left ventricular; RVEF, right ventricular ejection fraction; RV, right ventricular; LA, left atrium; ECV, extracellular volume; MOLLI, modified look-locker inversion recovery technique; indexed, divided by body surface area*.

The presence of myocardial fibrosis, independent of severity, was associated with increased left atrial volume (*p* = 0.02) but was not associated with any other CMR measurement. When compared with the other cardiac modalities, the presence of myocardial fibrosis was not associated with other cardiac measures assessed in this study.

The amount of myocardial fibrosis correlated with the following measurements on CMR: LVEF (Spearman correlation *r* = −0.55, *p* = 0.005) and LV end systolic volume (*r* = 0.50, *p* = 0.01), but did not correlate with any other CMR measurement.

When comparing participants older and younger than 50 years, there was no significant difference in the incidence of myocardial fibrosis (Figure 1A), also when considering the level of fibrosis (Spearman correlation *r* = 0.01, *p* = 0.96).

### Duchenne Muscular Dystrophy vs. Becker Muscular Dystrophy

When comparing women with a pathogenic variant associated with a DMD (*n* = 33) vs. BMD (*n* = 20) phenotype, we found that the presence of myocardial fibrosis on CMR was more frequent in carriers of DMD (61 vs. 28%, *p* = 0.02) (Figure 1B), but on echocardiography, there was no difference between the two groups in LVEF, diastolic dysfunction, LV dilatation, or longitudinal function. ECGs showed no difference in DMD-specific changes, incomplete RBBB, and Holter monitoring disclosed no difference in SVT or frequent VPCs between the two groups.

## Discussion

The present study demonstrates that women with dystrophin gene aberrations have a high prevalence of cardiac involvement that affects structure, as apparent from myocardial fibrosis on CMR (49%), function, as assessed by echocardiography (62%), and conduction abnormalities as demonstrated by ECG (72%) and Holter monitoring (43%).

On CMR, a new finding in our study was that myocardial fibrosis correlated with an increase in LV end systolic volume, further substantiating that fibrosis is associated with systolic dysfunction of the left ventricle in the carriers. CMR is more sensitive than echocardiography when investigating ventricular dimensions (25), giving us a more accurate measure of these structural changes. The frequency of myocardial fibrosis in the whole cohort (49%), and of women carrying *DMD* variants that predict DMD (61%) and BMD (28%), agrees with findings in other studies (DMD 35–65%, BMD 19–20%) (6, 10, 26). The frequency of fibrosis in pathogenic *DMD* variant carriers is as high as that seen in males affected by DMD (47–67%), but the amount of fibrosis is lower (3.8% in carriers vs. 8.1% in males) (26, 27). We also found that 35% had LV dilatation, and 10% had LV hypertrophy on CMR, which are higher frequencies than previously reported in women with pathogenic DMD variants (4, 7, 9). The level of myocardial fibrosis correlated with a decrease in LVEF, confirming earlier CMR studies in female carriers (6, 12, 28).

Cardiac dysfunction on echocardiography was found in 33 (62%) of women with a pathogenic *DMD* variant, which is considerably higher than reported in recent carrier studies (10). This increase is likely due to global longitudinal strain, which we investigated in the DMD carriers and which has not been studied before in this population. The measurement was reduced in 29 (63%) participants on echocardiography. Global longitudinal strain is a measurement of left ventricle systolic and diastolic function and is a long-term risk-predictor of cardiac morbidity and mortality (29). It reveals systolic dysfunction earlier than previous measurements, e.g., fractional shortening, providing us with a greater understanding of cardiac status and prognosis. Earlier studies have used fractional shortening to assess global function (4, 5, 9, 30). This measurement is one dimensional and less sensitive.

We found a high prevalence of DMD-specific ECG changes in our cohort. Earlier studies have shown a large difference in prevalence of both specific and general ECG changes, ranging from 7 to 47% (7–9, 11). The association between conduction abnormalities and myocardial fibrosis on CMR and left ventricular function has not been investigated before. In this study, we found no association between conduction abnormalities and ventricular function on echocardiography as well as myocardial fibrosis. These results agree with Thrush et al. (31, 32), who found no relationship between DMD-specific ECG changes and development of dilated cardiomyopathy in boys with DMD/BMD.

When examining the biomarkers, 49% of the cohort had elevated CK-MB levels. Both CK and CK-MB values have been seen to rise during both skeletal and heart muscle damage. A CK-MB/CK ratio <3% has been shown to predict that the rise likely relates to damaged skeletal muscle, rather than heart involvement (33). As we had no participants with a CK-MB/CK ratio ≥3%, we found no connection between an elevated ratio and cardiac affection in this group, which was also found by Hoogerward et al. (34).

Troponin and pro-BNP were only mildly elevated in some carriers, and the plasma levels of these two biomarkers did not associate with myocardial fibrosis on CMR. Troponin and proBNP have been suggested as good cardiac markers in myopathic patients (12, 35, 36), but these studies had <25 subjects or investigated only DMD patients and not carriers. Our study suggests that TnT and pro-BNP are unsuitable to indicate cardiac involvement in women with pathogenic *DMD* variants. This is supported by Hoogerwaard et al. (34) and Schade van Westrum et al. who also found evidence that troponin and proBNP do not predict cardiomyopathy in women with *DMD* variants (37).

Because of the wide range of cardiac modalities investigated in our study, we had the opportunity to compare the relationship between the different cardiac parameters. When investigating if one measurement, e.g., EF < 54%, was associated with other abnormal measurements, we found no significant association between any of the following: EF < 54%, diastolic dysfunction and dilatation on echocardiography, IRBBB and any DMD-specific change on ECG, couplets/triplets, SVT and frequent VPCs on Holter, and fibrosis on CMR. This indicates that each modality cannot, with any certainty, predict the presence of abnormalities in the other three modalities, hence, underlining the importance of a full cardiac workup for all carriers of pathogenic *DMD* variants, including CMR, at least once to give a complete picture of cardiac status.

The differences in cardiac involvement among carriers can be influenced by the level of skewed X chromosome inactivation. Some studies report a good correlation between clinical symptoms and skewed X chromosome inactivation, in particular, the relationship between skewed X chromosome inactivation and cardiomyopathy (38, 39). However, other studies contradict this theory (5, 40), including a recent study by Brioschi et al. (41) that, instead, suggested a possible relationship between dystrophin expression and symptoms. In the abovementioned studies, skewed X chromosome inactivation in carriers was mainly investigated in skeletal muscle biopsies or lymph node biopsies, and not in cardiac biopsies. The level of skewed X chromosome inactivation is known to differ among tissues (42). Since the level of X chromosome inactivation has not been studied in heart tissue of *DMD* variant carriers, the relationship between this and cardiac involvement is still unresolved.

It is inherently difficult to detect differences between women with *DMD* variants predicted to associate with DMD vs. BMD because the abovementioned skewed X chromosome inactivation obscures the results. In this study, we did, however, find a significantly higher frequency of cardiac fibrosis in the carriers of a pathogenic variant associated with DMD vs. BMD. However, we did not find the same difference between carriers of *DMD* variants predicted to give DMD vs. BMD for any of the other cardiac measurements we performed.

Our study does not provide longitudinal data, and we can therefore not predict if cardiac dysfunction progresses with time, as suggested by follow-up reports (11, 30). In our study, age did not correlate with LVEF or myocardial fibrosis. When comparing participants older and younger than 50 years, no significant difference was seen in systolic function, diastolic function, or myocardial fibrosis. The only significant difference was found in SVT and couplets/triplets on Holter monitoring, which are measurements normally seen more frequently with age. This lack of correlation between cardiac abnormalities and age was also found in a recent retrospective study made by McCaffrey et al. (4) in women with pathogenic *DMD* variants and other smaller cross-sectional studies of this group showing the same tendency (26, 43). This can be explained by the fact that our cohort represents a population with many different levels of skewed X chromosome inactivation, thus, concealing the effect of age on cardiac symptoms.

There is a possible selection bias concerning recruitment, as information about cardiac status is missing for the group of non-responders. The possibility that these women regarded themselves as being either asymptomatic or too symptomatic to participate must be considered and might skew the results in either direction. We have, however, recruited our subjects mainly from a genetic register and not through cardiac outpatient clinics. This is at variance with some of the earlier studies that have been retrospective (4–6) and recruited symptomatic patients from neurology clinics (5, 12, 43). From the sample of 20 interviewed non-participants, no one reported cardiac or neurologic symptoms or disease as a reason not to participate, and there was no difference in reported cardiac symptoms in the interviewed group compared with our cohort. We therefore believe that our cohort is representative for carriers of *DMD* variants.

Holloway et al. found that life expectancy and death from cardiac disease were not increased in a population of 94 women considered as definite carriers of pathogenic *DMD* variants compared with the general population (44). They therefore suggested that routine cardiac surveillance of carriers is unnecessary. In clinical experience, the morbidity and mortality for carriers is considered low. This, together with the findings of Holloway et al., indicates that the clinical significance of the findings in our study may not be as alarming as the frequency of cardiac dysfunction suggests.

The American Academy of Pediatrics estimates that 10% of women with pathogenic variants in *DMD* will progress to overt cardiomyopathy and recommends treating the women with evidence of cardiac involvement similar to DMD/BMD patients (45). Our study suggests that this number might be higher, and further longitudinal studies are necessary to investigate the development of cardiac affection in this group. Furthermore, randomized studies should be made to clarify the effects of treatment and the appropriate time to initiate treatment in this population.

In conclusion, the present study shows that cardiac involvement is found in over 2/3 of women with pathogenic variants in *DMD* that affects both structure and function of the heart. Systolic and diastolic function as well as myocardial fibrosis is affected independent of age, making early cardiac examinations and follow-up important. The study supports systematic screening of this group, including ECG, Holter, and cardiac imaging, so that symptoms related to pathogenic variants in *DMD* can be recognized, and relevant treatment can be initiated early.

## Data Availability Statement

The raw data supporting the conclusions of this article will be made available by the authors, without undue reservation.

## Ethics Statement

The studies involving human participants were reviewed and approved by Danish Data Protection Agency and the National Committee of Health Research Ethics. The patients/participants provided their written informed consent to participate in this study.

## Author's Note

Skeletal muscle investigations performed on the same cohort are presented in the simultaneously submitted article “Quantitative Muscle MRI and Clinical Findings in Women with Pathogenic Dystrophin Gene Variants” by Freja Fornander.

## Author Contributions

JV was responsible for the study design. TS, FF, NP, and MD collected the data. TS, JV, HB, AR, and RM contributed to data analysis. TS drafted the manuscript. All authors provided critical feedback and helped shape the research, the data interpretation, and the manuscript.

## Conflict of Interest

The authors declare that the research was conducted in the absence of any commercial or financial relationships that could be construed as a potential conflict of interest.

## Publisher's Note

All claims expressed in this article are solely those of the authors and do not necessarily represent those of their affiliated organizations, or those of the publisher, the editors and the reviewers. Any product that may be evaluated in this article, or claim that may be made by its manufacturer, is not guaranteed or endorsed by the publisher.

## Abbreviations

AFL/AF: atrial flutter/fibrillation
AVB: atrioventricular block
BMD: Becker muscular dystrophy
CK: creatine kinase
CK-MB: creatine kinase MB
CMR: cardiac magnetic resonance imaging
DMD: Duchenne muscular dystrophy
EF: ejection fraction
ECV: extracellular volume
GLS: global longitudinal strain
ILBBB: incomplete left bundle branch block
IRBBB: incomplete right bundle branch block
LBBB: left bundle branch block
LGE: late gadolinium enhancement
LV: left ventricular
LVEF: left ventricular ejection fraction
MOLLI: modified look-locker inversion recovery technique
MRC: Medical Research Council scale for muscle strength
NSVT: non-sustained ventricular tachycardia
proBNP: pro-brain natriuretic peptide
RBBB: right bundle branch block
SVPC: supraventricular premature contractions
SVT: supraventricular tachyarrhythmias
TnT: troponin T
VPC: ventricular premature contractions.
